# Systematic design for trait introgression projects

**DOI:** 10.1007/s00122-017-2938-9

**Published:** 2017-06-24

**Authors:** John N. Cameron, Ye Han, Lizhi Wang, William D. Beavis

**Affiliations:** 1 Department of Agronomy, Iowa State University, Ames, IA 50010 USA; 2 Department of Industrial and Manufacturing Systems Engineering, Iowa State University, Ames, IA 50010 USA

## Abstract

**Key message:**

**Using an Operations Research approach, we demonstrate design of optimal trait introgression projects with respect to competing objectives.**

**Abstract:**

We demonstrate an innovative approach for designing Trait Introgression (TI) projects based on optimization principles from Operations Research. If the designs of TI projects are based on clear and measurable objectives, they can be translated into mathematical models with decision variables and constraints that can be translated into Pareto optimality plots associated with any arbitrary selection strategy. The Pareto plots can be used to make rational decisions concerning the trade-offs between maximizing the probability of success while minimizing costs and time. The systematic rigor associated with a cost, time and probability of success (CTP) framework is well suited to designing TI projects that require dynamic decision making. The CTP framework also revealed that previously identified ‘best’ strategies can be improved to be at least twice as effective without increasing time or expenses.

**Electronic supplementary material:**

The online version of this article (doi:10.1007/s00122-017-2938-9) contains supplementary material, which is available to authorized users.

## Introduction

Trait introgression (TI) has been used for decades to transfer simply inherited traits from one cultivar to another. For example, native alleles for disease resistance and height were transferred through backcrossing in the “green revolution” (Swaminathan 2009) and trait introgression will continue to be the primary means of delivering novel single gene traits to the farmer. Indeed, the market impacts of single gene traits, such as transgenes for seed composition, insect resistance and herbicide resistance, have prompted development of high throughput technologies such as robotic seed chipping (Deppermann and Petersen 2009) for this purpose. Valuable single gene traits are being developed with biotechnologies, and are being discovered in wild and exotic germplasm (Kumar et al. 2010; Leung et al. 2015; Wang et al. 2015a, b).

Because the recurrent parent (RP) in a TI project is often a cultivar that has proven successful, a high level of genetic similarity to the RP is desired in introgression lines (ILs). Since the emergence of marker-assisted breeding, marker-assisted single gene TI projects have been investigated extensively (Hillel et al. 1990; Chevalet and Mulsant 1992; Visscher et al. 1996; Charcosset 1997; Frisch et al. 1999; Hospital 2001; Herzog and Frisch 2011; Peng et al. 2014; Herzog et al. 2014). The primary challenge in a TI project is to recover the genome of the RP when segments of the donor chromosome surrounding the desirable allele, referred to herein as an *event locus*, are retained due to linkage (Hanson 1959; Young and Tanksley 1989; Stam and Zeven 1981; Naveira and Barbadilla 1992). Thus, one of the goals of TI projects includes minimizing the amount of non-recurrent parent (NRP) genome especially in regions flanking the event locus. The goal of minimizing the size of the genomic region adjacent to the event locus, also known as minimizing linkage drag through selection (LDS), is accomplished through use of informative markers closely linked to the event locus. Additional goals of a TI project include minimizing the numbers of progeny, numbers of marker assays and numbers of generations required to minimize the NRP (Herzog and Frisch 2011; Peng et al. 2014; Herzog et al. 2014).

The trade-offs among minimizing generations, numbers of progeny, numbers of markers and amount of NRP represent an optimization challenge. Indeed the title of a motivating publication by Peng et al. (2014) suggested that TI could be approached as an optimization challenge. Peng et al. (2014) stated that their primary objective was to “…identify optimal breeding strategies for MTI [marker trait introgression] using computer simulation, focusing on efficiencies for single event introgression…”. They suggested a measurable metric for selected ILs with an average residual NRP of less than or equal to 8 cM and no more than an average of 1 cM of foreground region from non-recurrent parent (FRNRP), i.e., the NRP linked to the desirable allele at the event locus. Although they did not frame the challenge using an objective function involving time, cost and probability of success (CTP), their approach had specific objectives that could be translated into a mathematical objective function with decision variables and constraints for the parameters. The specific objectives of Peng et al. (2014) can be stated as: Identify breeding strategies to produce avg(NRP) ≤8 cM, and avg(FRNRP) ≤1 cM with the fewest marker assays, and fewest number of progeny in five backcross generations. The statement represents a multi-objective function where avg(FRNRP) and avg(NRP) are considered parameters and number of marker assays per plant, number of plants per generation and breeding strategies are considered decision variables.

Herein, we revisit optimization of single gene TI by Peng et al. (2014) using the CTP framework from Operations Research (OR). The CTP framework enables decision makers to quantify the trade-offs between maximizing probability of success and minimizing costs and time using Pareto Optimal graphics. Explicitly, we are not suggesting novel selection strategies, rather we demonstrate that existing selection strategies can be twice as effective for less cost and time by merely framing the objectives as a mathematical optimization (CTP) model. Pareto optimality also enables the researcher to decide whether limited efforts have any chance of success. Sometimes it is better to do nothing than to waste resources on a project that has little chance of success.

## Materials and methods

### Objectives and metrics

In the CTP framework, both numbers of progeny (*np*) and markers assays contribute to costs. Costs can be calculated on a cost per plant basis, then multiplied by total number of plants required by the breeding strategy over generations. Time in the CTP framework is represented by the number of generations, which also contributes to the total costs of the breeding strategy. In an operational TI project, the number of generations will depend on whether selection criteria of a breeding strategy are met in any given generation. Thus, for the CTP framework we treated number of generations as dependent on a dynamic decision process. The selection criteria of the breeding strategies in this manuscript determine both numbers of markers required, and the generation that a simulation finishes. Perhaps most importantly the CTP framework requires the researcher to rigorously define success. Rather than report average values for an unknown multivariate distribution, we defined success as outcomes in which the final four selected individuals had ≤1 cM of the donor alleles adjacent to the event locus (*el*) and ≤8 cM of donor alleles throughout the rest of the genome. This enabled us to determine the probabilities of success [*P*(s)] associated with such outcomes for each breeding strategy.

### Definitions and notation

Let *l* represent the total number of discrete loci in the genomes of Donor and Recurrent homozygous lines where both *G*_*D*_ and *G*_*R*_ genomes are members of the same taxonomic group with genome *G,* i.e., $l \subset \{ G_{D} \cup G_{R} |G_{D} \cup G_{R} \subset G,G_{D} \cap G_{R} \not\subset \emptyset \} .$ For purposes of illustration, we adopt a maize-like genome, (*G*) considered by Peng et al. (2014) where *G* is organized as 10 linkage groups, each consisting of genetic length = 179 cM.

**Fig. 1:**
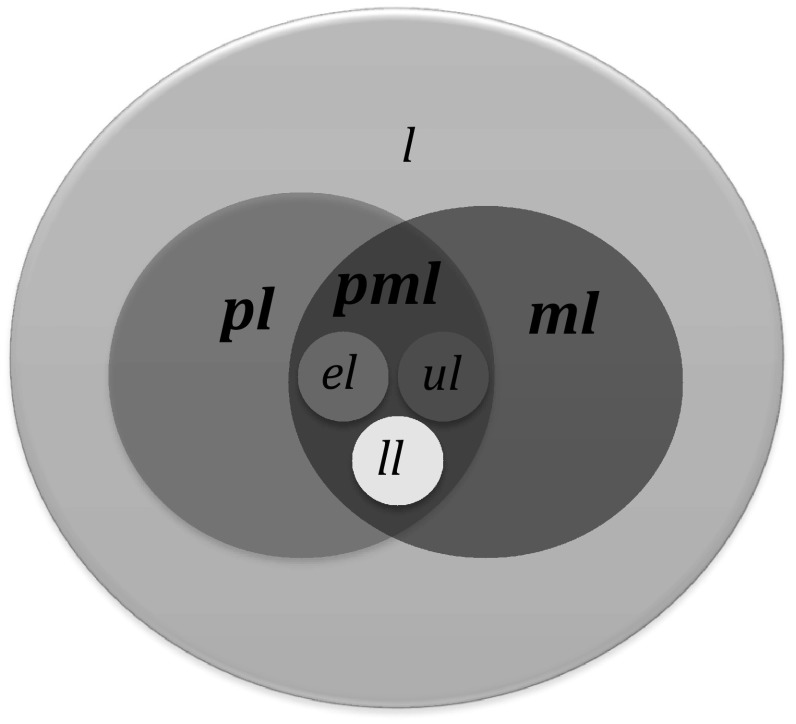
Representation of the relation between loci (*l*), loci with polymorphic alleles (*pl*), marker loci (*ml*), marker loci with polymorphic alleles (*pml*), and three unique subsets (*el*, *ll*, *ul*) of marker loci with polymorphic alleles that are used for selection

From these basic definitions for representing the loci in donor and recipient genomes *G*_*D*_ and *G*_*R*_, we let (Fig. 1):

- loci with polymorphic alleles (*pl*) represent a subset of *l*, i.e., $pl \subset l$;
- ten non-overlapping subsets of 180 *pl* are genetically located at 1 cM intervals on each linkage group;
- marker loci (*ml*) represent a subset of *l* that can be assayed by a marker technology, i.e., $ml \subset l$;
- marker loci with polymorphic alleles (*pml*) represent the subset at the intersection of *ml* and *pl*, i.e., $pml \equiv ml \cap pl$;
- *el* represents the event locus in *G*_*D*_ with the desired donor allele, where $el \in pml$;
- *ll* represent 20 tightly linked *pml* spaced in 1 cM intervals from *el*, $ll \subset pml,\text{ }ll \cap el \subset \emptyset$;
- *ul* represent loci that are not tightly linked to *el* and are distributed uniformly throughout G, $ul \subset pml,\text{ }ul \cap el \subset \emptyset ,\text{ }ul \cap ll \subset \emptyset$;
- *nma/p* denote the number of marker assays (*nma*) per sampled plant (*p*), where each marker assay (*ma*) assesses the genotypes at a set of *pml*.

Next consider genotypes at subsets of loci in diploid or allopolyploids genomes where there will be two possible alleles at each locus: one from the donor and one from the recipient. If alleles at each locus are compared there are two possible outcomes: the alleles are the same, i.e., monomorphic, or the alleles are not the same, i.e., polymorphic and informative. Based on these considerations, we can represent genotypes at subsets of loci as matrices by letting:

- **pl**_1800,2_ represent a (1800 × 2) matrix of genotypes at *pl*.
- **el** represent a (1 × 2) matrix of the genotypic values at *el*.
- **ll**_20,2_ represent a (20 × 2) matrix of genotypic values at the 20 *ll*.
- **ul**_*a*,2_ represent a (*a* × 2) matrix of genotypes at *pml* that are not tightly linked to *el*.
- *a* will be the sum of *pml* on nine linkage groups that segregate independently of the linkage group with *el* and the set of *pml* on the linkage group that contains *el*, excluding any *pml* that are also members of *ll* and *el*. Explicitly *a* will be either 98 or 187 depending on the selection strategy.

If we let desirable alleles in **pl**, **el**, **ll** and **ul** = 1 and undesirable alleles = 0, then

- ${\mathbf{J}}_{1800,2}^{pl} ,\,{\mathbf{J}}_{1,2}^{el} ,\,{\mathbf{J}}_{20,2}^{ll} ,\,{\mathbf{J}}_{a,2}^{ul}$ are matrices consisting of only desirable alleles in **pl, el**, **ll** and **ul**.

### Simulated genomic and marker loci

Simulated polymorphic loci (*pl*) were spaced in 1 cM intervals, from position 0 to position 179, for a total of 180 per linkage group. For purposes of selection, we simulated the event locus marker on the first linkage group at position 90, i.e., 89 cM from the nearest end. We also positioned 10 *ll* ranging from 79 to 88 cM on one side of the event, and 90 to 99 cM on the other side. The spacing of *ul* markers on the first linkage group for strategies 1–3 included: a marker placed at 0, 19, 39, 59, 119, 139, 159 cM, and an end marker at 179 cM. Similarly, the spacing of *ul* markers for strategies 4–6 included: a marker placed at 0, 9, 19, 29, 39, 49, 59, 69, 109, 119, 129, 139, 149, 159, 169 cM and an end marker at 179 cM. On each of the remaining nine linkage groups, additional *ul* markers were placed at 79 and 99 cM for strategies 1–3, and 79, 89, and 99 cM for strategies 4–6. Therefore, the total number of *ul* markers used in strategies 1–3 was 98, and the total number of *ul* markers used in strategies 4–6 was 187.

### Selection criteria

We adopted the notation for selection criteria provided by Peng et al. (2014):

- ES refers to selection based on presence of the desirable allele at the *el*.
- LDS refers to selection for the desirable alleles at *ll*. A weighting vector was used to assign recombination events occurring closer to the *el* a higher value than those farther away. The weighting vector for one side of the event locus (Peng et al. 2014) was: $\left[ {\frac{10}{55},\frac{9}{55},\frac{8}{55},\frac{7}{55},\frac{6}{55},\frac{5}{55},\frac{4}{55},\frac{3}{55},\frac{2}{55},\frac{1}{55}} \right]$, with $\frac{10}{55}$ indicating the weight of the closest *ll*, and $\frac{1}{55}$ indicating the weighting of the *ll* farthest from the *el*. The sum of the weighting vector is 1; thus, the maximum FR score an individual can take in any generation is 1. The binary allele present at each member of **ll**, for each individual, was multiplied by the corresponding value in the weighting vector and the products were summed. The progeny with the highest scores were selected and advanced.
- RPS refers to selecting for the desirable alleles at *ul*.

To develop dynamic decision rules to apply to progeny from backcross generations, we note that if the genotypic scores in any generation are **el** = **J** ^*el*^, or **ll** = **J** ^*ll*^, or **ul** = **J** ^*ul*^, then the respective ES, LDS or RPS in subsequent generations would represent unnecessary and wasteful activities. We designate the generation when selection criteria are met, i.e., **el** = **J** ^*el*^, **ll** = **J** ^*ll*^ and **ul** = **J** ^*ul*^ as threshold generations *t* ^*el*^, *t* ^*ll*^ and *t* ^*ul*^, respectively. We designate *t*′ as a terminal generation for any selection strategy. In this generation, **ul** = **J** ^*ul*^ in each of the final selected individuals. Seed with **el** = **J** ^*el*^ will be obtained after selfing each of these final lines; however, our simulation stops when **ul** = **J** ^*ul*^; we do not count the extra generation for selfing. Thus, *P*(*t*′ ≤ *g*) represents the probability that the terminal generation is reached before generation *g* + 1.

### Selection strategies

Three selection strategies previously investigated by Peng et al. (2014) were used to demonstrate the impact of the CTP framework. We also investigated the impact of doubling the *nma* suggested by Peng et al. (2014) for the *ul*. The resulting selection strategies and *nma* for selection criteria are indexed by *k* (for *k* = 1–6) (Table 1).

**Table 1:** Algebraic notation for selection strategies and number of markers (*nma*) assayed on each individual, for each selection criterion, per generation

*k*	Strategy_*k*_	*nma* (ES, LDS, RPS)
1	$({\text{ES + LDS}})_{{\forall g \in \text{ }\{ g \le 2, \ne {\text{t}}^{el} , \ne {\text{t}}^{ll} \} }} ({\text{ES + RPS}})_{{\forall g \in { \{ }g > 2, \ne {\text{t}}^{el} , \ne {\text{t}}^{ul} \} }}$	1, 20, 98
2	$({\text{ES + LDS}})_{{\forall g \in \text{ }\{ g \le 3, \ne {\text{t}}^{el} , \ne {\text{t}}^{ll} \} }} ({\text{ES + RPS}})_{{\forall g \in { \{ }g > 3, \ne {\text{t}}^{el} , \ne {\text{t}}^{ul} \} }}$	1, 20, 98
3	$({\text{ES + LDS + RPS}})_{{\forall g \in {\{ }g > 0, \ne {\text{t}}^{el} , \ne {\text{t}}^{ll} , \ne {\text{t}}^{ul} \} }}$	1, 20, 98
4	$({\text{ES + LDS}})_{{\forall g \in \{ g \le 2, \ne {\text{t}}^{el} , \ne {\text{t}}^{ll} \} }} ({\text{ES + RPS}})_{{\forall g \in {\{ }g > 2, \ne {\text{t}}^{el} , \ne {\text{t}}^{ul} \} }}$	1, 20, 187
5	$({\text{ES + LDS}})_{{\forall g \in \{ g \le 3, \ne {\text{t}}^{el} , \ne {\text{t}}^{ll} \} }} ({\text{ES + RPS}})_{{\forall g \in {\{ }g \ge 3, \ne {\text{t}}^{el} , \ne {\text{t}}^{ul} \} }}$	1, 20, 187
6	$({\text{ES + LDS + RPS}})_{{\forall g \in {\{ }g > 0, \ne {\text{t}}^{el} , \ne {\text{t}}^{ll} , \ne {\text{t}}^{ul} \} }}$	1, 20, 187

Explicitly,

*k* = 1 represents ES + LDS for two initial backcross generations, followed by ES + RPS for as many backcross and self-pollination generations as needed for *ul* to become homozygous for the RP alleles in four ILs.

*k* = 2 represents ES + LDS for a maximum of three initial backcross generations followed by ES + RPS for as many backcross generations as needed for 98 *ul* to become homozygous for the RP alleles in four ILs. If the selection criteria for ES + LDS are met after two generations, then ES + RPS begins in generation 3.

*k* = 3 represents ES + LDS + RPS for as many backcross generations as needed for 20 *ll* and 98 *ul* to become homozygous for RP alleles in four ILs. If the *ll* markers become fixed in any generation, then LDS selection is no longer employed and ES + RPS is carried out until all *ul* markers are fixed for the RP alleles.

*k* = 4 represents ES + LDS for two initial backcross generations followed by ES + RPS for as many backcross generations as needed for 187 *ul* to become homozygous for the RP alleles in four ILs. This strategy is analogous to strategy 1, the only difference being that a denser set of *ul* markers are used in RPS selection.

*k* = 5 represents ES + LDS for a maximum of three initial backcross generations followed by ES + RPS for as many backcross generations as needed for 187 *ul* to become homozygous for the RP alleles in four ILs. If the selection criteria for ES + LDS are met after two generations, then ES + RPS begins in generation 3. This strategy is analogous to strategy 2, the only difference being that a denser set of *ul* markers are used in RPS selection.

*k* = 6 represents ES + LDS + RPS for as many backcross generations as needed for 20 *ll* and 187 *ul* to become homozygous for RP alleles in four ILs. If the *ll* markers become fixed in any generation, then LDS selection is no longer employed and ES + RPS is carried out until all *ul* markers are fixed for the RP alleles. This strategy is analogous to strategy 3, the only difference being that a denser set of *ul* markers are used in RPS selection.

As per Peng et al. (2014), ES was always carried out first, culling all individuals without the event. In strategies where two selection criteria were implemented (i.e., ES + RPS or ES + LDS), four individuals were selected after ES culling to backcross to the RP, based on either the highest score for desirable alleles in the *ll* set or highest number of desirable alleles in *ul,* respectively. In strategies using all three selection criteria (i.e., ES + LDS + RPS), eight individuals with the highest score for desirable alleles in the *ll* set were selected from among those with the desirable allele at the *el*, and subsequently four of the eight with the largest number of desirable alleles in the *ul* set were selected to backcross the RP.

The subscript *t* in each set of selection criteria within a strategy indicates the terminal generation based on the selection criteria. The selection criteria within a strategy were implemented sequentially, with the first set of criteria carried out until generation *t* is completed, followed by generations of selection on the second set of criteria until generation *t* indicated by the second subscript, etc.

For purposes of comparison, we also investigated a fixed number of five backcross generations as per Peng et al. (2014). Explicitly, estimation of *P*(*t*′ ≤ *g*), *P*(s) and costs in this situation were not based on the total number of generations needed until **el** = **J** ^*el*^, **ll** = **J** ^*ll*^ and **ul** = **J** ^*ul*^. Rather, they were based on the selection criteria applied to five backcross generations; no more, no less. For the sake of comparisons and notational convenience, we label the six strategies that were based on a fixed number of five backcross generations as *k* = *n*′, e.g., *k* = 1′ represents breeding strategy 1, but without dynamic decision-making.

### Objective model

**M1**:

1 $$\hbox{min} \;\zeta = c_{k}^{p} + c_{k}^{m} ,$$

2 $$\hbox{min} \;g ,$$

3 $$\hbox{min} \; P{(\rm s)},$$

4 $$\begin{array}{l} {\text{s}} . {\text{t}} .\hfill \\ c_{k}^{m} = \sum\limits_{g = 1}^{{t^{el} ,t^{ul} }} {\{ [(\$ 0.05/ma)(nma_{g} /p) + \$ 0.50/p][np_{g} ]|{\text{strategy}}_{k} \} ,} \hfill \\ \end{array}$$

5 $$c_{k}^{p} = \sum\limits_{g = 1}^{{t^{el} ,t^{ul} }} {[(\$ 5.00/p)_{g} (np_{g} )|{\text{strategy}}_{k} ]} ,$$

6 $${\text{Strategy}}_{k} \in \left\{ \begin{aligned} & ({\text{ES + LDS}})_{{\forall g \in \{ g \le 2, \ne t^{el} , \ne t^{ll} \} }} ({\text{ES + RPS}})_{{\forall g \in {\{ }g > 2, \ne t^{el} , \ne t^{ul} \} }} ; \\ & ({\text{ES + LDS}})_{{\forall g \in \{ g \le 3, \ne {\text{t}}^{el} , \ne {\text{t}}^{ll} \} }} ({\text{ES + RPS}})_{{\forall g \in {\{ }g \ge 3, \ne t^{el} , \ne t^{ul} \} }} ; \\ & ({\text{ES + LDS + RPS}})_{{\forall g \in {\{ }g > 0, \ne t^{el} , \ne t^{ll} , \ne t^{ul} \} }} \ldots \\ \end{aligned} \right\},$$

7 $$np_{{g \ne t^{el} ,t^{ll} ,t^{ul} }} \in \{ 100,200,300, \ldots 1000\} ,$$

8 $$(nma_{{g \ne t^{el} ,t^{ll} ,t^{ul} }} /p) \in \{ 21,99,119,188,208|{\text{strategy}}_{k} \} ,$$

9 $$s = \{ {\mathbf{ll}}_{,1} \bullet {\mathbf{ll}}_{,2} \ge 19 \cap \text{ }{\mathbf{pl}}_{{{\mathbf{,1}}}} \bullet {\mathbf{pl}}_{{{\mathbf{,2}}}} \ge 1792\} ,$$

10 $$P({\text{s}}) = {\text{freq}}(s|{\text{strategy}}_{k} ,np_{g} ).$$

The objectives of **M1** are to minimize the cost of carrying out the TI program (1), in as few generations possible (2) and maximize the probability of success (3). Costs of labor and materials for nursery activities of planting, growing, cultivating, and manually crossing and harvesting plants are represented by *c* ^*p*^. Laboratory costs associated with tissue sampling, transportation of tissues, extraction of DNA and marker assays from sampled plants are represented by *c* ^*m*^. Calculation of costs of marker assays (4) is based on the tape array marker technology that permits the breeder to select *ma* for *el*, *ll* and two densities of *ul* at a cost of about $0.05 per *ma*, which does not represent the total costs for *ma* because there are also costs associated with tissue sampling, DNA isolation and shipping DNA to a lab responsible for conducting the assays. Currently, these costs are about $0.50 per sample. We also used a cost estimate of $5.00 per plant for nursery operations (5). Total costs depended on the number of generations required for the selection strategies (6), *np* per generation (7), and *nma* per progeny (8) for the *el, ll* and *ul.* Note that the number of generations required will be variable depending upon which generation selection criteria are met for the set of *el*, *ll* or *ul* used in each selection strategy. Success is defined as development of at least four ILs with no more than 8 cM of NRP and no more than 1 cM of FRNRP. Mathematically, this was represented as (9): marker loci *ll*, that are tightly linked to *el,* need to be homozygous for the recurrent parent at all but one of the *ll*. In (9) **pl**_,1_ and **pl**_,2_ represent the first and second column vectors of **pl**. If the inner product of these two vectors for any individual is ≥1792, then the individual will have no more than 8 cM of the NRP, assuming that the probability of double recombination events within 1 cM intervals across all generations required to produce the individual is zero. The probability of success, *P*(s), described in (10), depends on the stochastic processes of transmission genetics. Since there are no known functional relationships between costs, time and success computer simulations were used for the stochastic processes underlying outcomes from the TI process for each of the strategies. The Pareto optimal plots for this model with respect to cost and *P*(s) are presented in Fig. 3. Figure 4 shows a modification of this model where time has been constrained to five BC generations.

### Simulation models

Two thousand simulations were carried out for each of the selection strategies on ten sample sizes (i.e., *np*) per generation, resulting in 20,000 simulations per strategy. The simulated genomes included ten linkage groups consisting of 180 genetic loci placed at 1 cM intervals on each. These genetic loci provided genotypic information for 1800 *pl* in **pl**, as well as genotypes for subsets of marker loci represented in **el**, **ll** and **ul**. Recombination between adjacent *pl* was simulated using Haldane’s mapping function. Simulated selection and crossings were based on the genotypes in **el**, **ll** and **ul** and the decision rules for continued backcrossing described in the selection strategies. An explicit description of the simulation model and implemented code, written in MATLAB 2015a, is available (Supplementary Appendix 1).

### Evaluation metrics

If marker selection criteria were met, then the generation in which the TI process was completed was recorded. Based on 2000 simulations for each combination of *np* and strategy, we estimated the frequency of meeting the selection criteria, *P*(*t*′ ≤ *g*). If the final four selected individuals from each combination were evaluated using **pl** and the conditions described in (9) were met for all four selected individuals, the simulation was recorded as a success. In this way, we were able to obtain estimates for *P*(s). The set of solutions representing trade-offs between *ζ* and *P*(s), also known as a Pareto frontier, was graphed to enable quantitative assessment among competing objectives.

### Cost calculations

The expected cost is weighted each generation by the probability that the selection criteria are met in that generation. Consider strategy *k* = 1: Because there were no simulated results indicating that the first set of selection criteria was met in less than three generations, the probability of assaying more than 21 markers for the first two generations was zero. Also the probability of assaying any number of markers other than 101 for subsequent generations is zero. Consider next strategy *k* = 2 which consists of three generations of (ES + LDS) followed by generations of (ES + RPS) until **el** = **J** ^*el*^, and **ul** = **J** ^*ul*^. For the larger samples of progeny, there is a greater probability that all the *ll* markers on either side of the *el* will be fixed for the allele from the RP after the second BC generation. In simulations where this occurred, the third BC generation was not used for (ES + LDS) selection, but rather (ES + RPS). Thus, we used empirically determined probabilities of meeting the selection criteria to weight the costs of the number of marker assays in each generation associated with each breeding strategy.

## Results

**Fig. 2:**
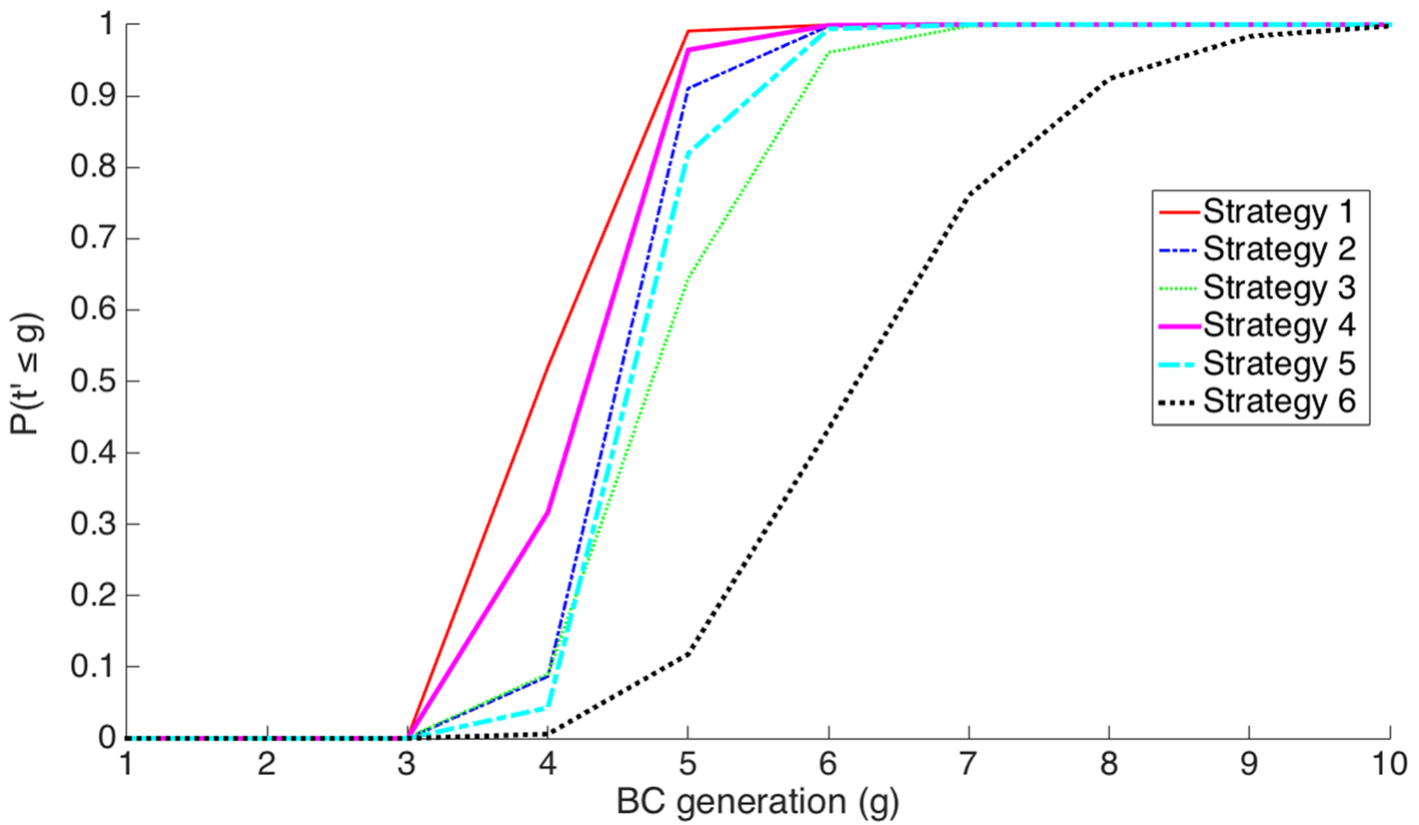
Estimated probability that selection criteria were met in *g* or fewer backcross generations, *P*(*t*′ ≤ *g*), for six *k* = *n* selection strategies in which 100 progeny were evaluated each generation

In simulations consisting of 100 progeny per backcross generation, the number of backcross generations required to meet the selection criteria ranged from as few as three generations in 1% of the simulations of strategy 1 to as many as 11 generations for 100% of the simulations of strategy 6 (Fig. 2). In general, the fewest generations were needed to meet the selection criteria using strategies 1 and 4, followed by strategies 2 and 5, while strategies 3 and 6 required the greatest number of generations to meet the selection criteria. As sample sizes increased, the total number of generations required to meet selection criteria in 100% of the simulations decreased (Supplementary Materials, Tables 1–6). For example, it took seven backcross generations for 100% of the simulations to meet the selection criteria of strategy 1 in samples consisting of 100 progeny per generation, while it only took four generations to meet the same criteria with sample sizes greater than 700.

**Fig. 3:**
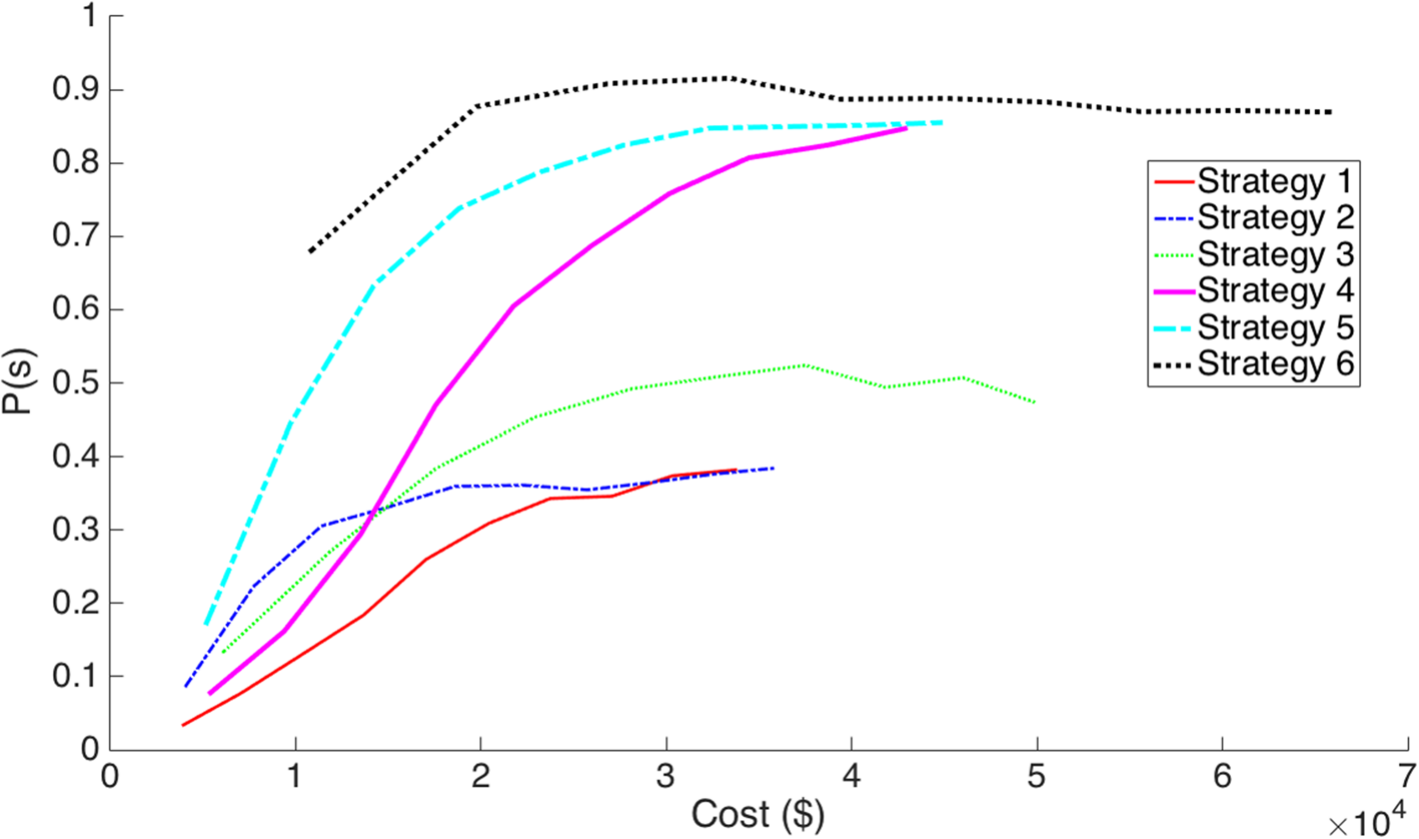
Probability of successfully meeting the breeding objectives, *P*(s), and expected costs for each of six *k* = *n* selection strategies

In contrast, the probability of success, *P*(s), after 100% of the simulations met the selection criteria, was greatest for strategy 6 and least for strategies 1 and 2 (Table 2). For example, using a sample size of 100 per generation *P*(s) after 100% of the simulations met the selection criteria for strategy 1 was 0.034, while it was 0.681 for strategy 6. In general, the *P*(s) after 100% of the simulations met selection criteria increased profoundly by doubling the number of marker assays used for the set of *ul* (strategies 4, 5 and 6 of Table 2). Also, the *P*(s) increased with sample size, although it approached an asymptote for all strategies indicating that there are limits to the *P*(s) based on increasing sample sizes (Fig. 3). For strategy 6, the asymptote was approached with 200 progeny per generation. Thus, strategy 6 provides the greatest probability of success for a cost of about $10,800. To spend less with strategies using 98 *ul* markers will result in less than half the success rate (Fig. 2). Note, however, that strategy 6 with 200 progeny per generation will require about eight or nine generations to provide high probability of success (Supplementary Materials and Table 6). Strategy 5 can achieve similar probabilities of success as strategy 6 in only five or six generations, but requires a cost that is about two and a half times as great using a sample size of about 700.

**Table 2:** Probability of successfully meeting the breeding objectives, *P*(s), with ten different sample sizes (*np*) evaluated for markers each generation, for six selection strategies

Strategy_*k*_	*np*_*g*_ = 100	*np*_*g*_ = 200	*np*_*g*_ = 300	*np*_*g*_ = 400	*np*_*g*_ = 500	*np*_*g*_ = 600	*np*_*g*_ = 700	*np*_*g*_ = 800	*np*_*g*_ = 900	*np*_*g*_ = 1000
*k* = 1	0.034	0.077	0.130	0.184	0.260	0.309	0.343	0.346	0.374	0.382
*k* = 2	0.088	0.223	0.305	0.331	0.360	0.361	0.355	0.364	0.376	0.384
*k* = 3	0.134	0.271	0.384	0.453	0.492	0.509	0.524	0.494	0.507	0.473
*k* = 4	0.078	0.162	0.294	0.471	0.605	0.688	0.759	0.807	0.825	0.847
*k* = 5	0.174	0.445	0.634	0.738	0.788	0.825	0.848	0.850	0.852	0.855
*k* = 6	0.681	0.877	0.909	0.916	0.887	0.888	0.883	0.870	0.872	0.870

The probability that selection criteria will be met in five or fewer backcross generations for each of the strategies indicates that strategies 1′, 2′, and 4′ have very high probability that the selection criteria will be met in five or fewer generations with as few as 100 progeny (Fig. 2). For selection strategies 3′ and 5′, there is better than 60% chance of meeting selection criteria using 100 progeny while strategy 6′ has a very low probability of meeting the selection criteria in five or fewer generations. For selection strategies 5′ and 6′, it requires about 600 progeny to be evaluated every generation to assure that the probability of meeting selection criteria in five or fewer generations is greater than 0.8.

**Fig. 4:**
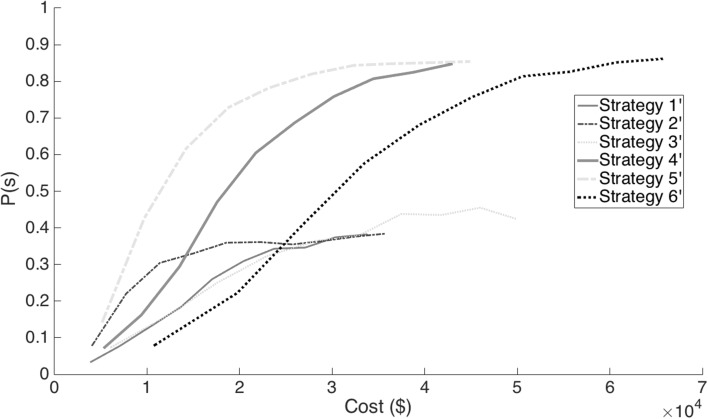
Probability of successfully meeting the breeding objectives in five BC generations, *P*(s), and expected costs for each of six *k* = *n*′ selection strategies

On the other hand, the probability of success, *P*(s), after five backcross generations, was greatest for strategy 5′ and least for strategy 1′ (Table 3). For example, using a sample size of 100 per generation after five generations strategy 1′ was estimated to be successful in only 7 of 2000 simulations, whereas strategy 5′ was successful in 14.5% of the simulations. In general, the probability of success increased profoundly by doubling the number of marker assays *nma* used for the set of *ul*, i.e., strategies 4′, 5′ and 6′ (Table 3). Also, the *P*(s) increased with sample size, although it approached an asymptote for all strategies indicating that there are limits to success based on increasing *np* (Fig. 4). In addition to a higher estimated *P*(s), strategies 4′ and 5′ have significant cost advantages relative to strategy 6′ for *np* >300. Peng et al. (2014) reported strategy 2′ as optimal. Strategy 5′ can achieve a *P*(s) of 0.844 with 700 progeny per generations for a cost of $29,245, whereas its analog strategy (strategy 2′) with a sparser density of *ul* markers has a *P*(s) of only 0.378 with 1000 progeny costing $32,942. Thus, strategy 5′ more than doubles the *P*(s) of strategy 2′ for less cost (Supplementary Materials and Figure 4).

**Table 3:** Probability of successfully meeting the breeding objectives in five BC generations, *P*(s), with ten different sample sizes (*np*_*g*_) evaluated for markers each generation, for six introgression strategies

Strategy_*k*′_	*np*_*g*_ = 100	*np*_*g*_ = 200	*np*_*g*_ = 300	*np*_*g*_ = 400	*np*_*g*_ = 500	*np*_*g*_ = 600	*np*_*g*_ = 700	*np*_*g*_ = 800	*np*_*g*_ = 900	*np*_*g*_ = 1000
*k*′ = 1′	0.034	0.077	0.130	0.184	0.260	0.309	0.343	0.346	0.374	0.382
*k*′ = 2′	0.080	0.220	0.304	0.330	0.360	0.361	0.355	0.364	0.376	0.384
*k*′ = 3′	0.074	0.155	0.250	0.322	0.364	0.380	0.438	0.435	0.455	0.424
*k*′ = 4′	0.074	0.162	0.294	0.471	0.605	0.688	0.759	0.807	0.825	0.847
*k*′ = 5′	0.145	0.427	0.617	0.729	0.782	0.820	0.844	0.848	0.851	0.854
*k*′ = 6′	0.080	0.223	0.412	0.576	0.681	0.757	0.814	0.826	0.852	0.862

## Discussion

Simulations of TI have been reported for about 20 years. Visscher et al. (1996) and Charcosset (1997) employed simulations to investigate the importance of genomic locations and number of marker assays on TI projects. Frisch et al. (1999) utilized simulation models to compare quantities of NRP genome based on the numbers of markers and sample sizes evaluated in early to late backcross generations. Frisch and Melchinger (2005) also explored a wide range of selection strategies using simulation models. However, these reports described exploratory research without explicit competing objectives.

Herzog and Frisch (2011) began to consider optimization of competing objectives in TI projects by comparing the distribution of recurrent parent genome in the 10% quantiles of progeny from a final backcross generation for fixed-cost high throughput (HT) marker technologies versus variable-cost single marker (SM) technologies for several backcross breeding strategies. Herzog et al. (2014) extended their approach to compare costs of creating 100 ILs using doubled haploid versus selfing schemes coupled to marker-assisted backcrossing strategies. As already noted, Peng et al. (2014) utilized simulations to investigate the ability of at least 50 breeding strategies to meet five competing objectives. Herein, we have demonstrated that the next logical step is to formalize objectives of TI projects into a mathematical function (M1) that can be optimized in the sense that trade-offs among competing objectives can be quantified and results can be represented as Pareto Optimal curves for use in decision making. Explicitly, we did not investigate novel selection strategies, rather we demonstrate that framing the objectives as a CTP model can reveal existing selection strategies may be more effective, cost less and take less time.

Although prior reports on optimization of TI projects (Herzog and Frisch 2011; Herzog et al. 2014; Peng et al. 2014) did not frame their TI challenges using an objective function, they did provide specific objectives that can be translated into an objective function with decision variables and constraints. For example, consider Peng et al. (2014). Their specific objectives were to identify breeding strategies that produced ILs with an avg(NRP) ≤8 cM, and avg(FRNRP) ≤1 cM with the fewest marker data points, and fewest number of plants grown, in no more than five backcross generations. These objectives can be represented in the following objective function:

11 $$\hbox{min} \;c_{k}^{p} ,$$

12 $$\hbox{min} \;c_{k}^{m} ,$$

13 $$\hbox{min} \;g,$$

14 $${ \hbox{min} }\;{\text{avg}}({\text{FRNRP}}),$$

15 $${ \hbox{min} }\;{\text{avg(NRP),}}$$

16 $$\begin{aligned} & {\text{s}} . {\text{t}} .\\ & c_{k}^{p} = \sum\limits_{g = 1}^{t} {(np_{g} |{\text{strategy}}_{k} ),} \\ \end{aligned}$$

17 $$c_{k}^{m} = \sum\limits_{g = 1}^{t} {((nma/p)_{g} (np_{g} )|{\text{strategy}}_{k} ),}$$

18 $${\text{Strategy}}_{k} \in \{ {\text{ES}}_{t = 5} ;({\text{ES }}{ + }{\text{LDS + RPS}})_{t = 5} ;({\text{ES + LDS}})_{t = 3} ({\text{ES }}{\text{ + RPS}})_{t = 5} ; \ldots \} ,$$

19 $$np_{g} \in \{ 80,20,50,100,200,400,600,800,1000,1500,2000\} ,$$

20 $$(nma/p)_{g} \in \{ 1,21,101,121\} ,$$

21 $$0 < t \le 5,$$

22 $${\text{avg(FRNRP)}} \le 1\,{\text{cM,}}$$

23 $${\text{avg(NRP)}} \le 8\,{\text{cM}} .$$

This objective function has five objectives represented by the first five statements, where *c* ^*m*^, *c* ^*p*^, *g*, avg(FRNRP) and avg(NRP) are considered parameters; marker assays per plant (*nma/p*), number of plants (*np*) and strategy in the next five statements are considered decision variables and the final three statements are constraints on the decision variables. It is possible that our understanding of their objectives, decision variables or constraints is not accurate and, thus, the objective function is not correct. The beauty of formalizing the objectives as an objective function is that there is no ambiguity in the objectives, decision variables and constraints. Thus, if our understanding of their text is not accurate, the mathematical statements in the objective function can be changed easily with the correct interpretation. Further, framing the objectives as an objective function enabled us to recognize the objectives in the CTP framework.

Using the CTP framework, we chose to define success as a discrete event rather than as average contributions of donor genome segments because decision makers need clear metrics to effectively communicate the trade-offs among objectives. For example, a significant probability of failure may be represented by strategies and *np* that produce final ILs in which the avg(FRNRP) ≤1 and avg(NRP) ≤8. If these averages are associated with a strategy in which the *P*(s) = 0.4 then assessment of risk is very different than if it is associated with a *P*(s) = 0.6. Another issue that arises with using averages of residual cM length NRP is that those data are not symmetrically distributed, and instead have long tails like a negative binomial distribution (data not shown). The probability of success *P*(s) concisely summarizes all the goals of the breeding program as a binomial response variable, allowing the breeder to compare strategies and resource allocations levels in meaningful way. Appropriate metrics are essential to the optimization of breeding programs, and using the *P*(s) as a method of comparison is a major advantage of this formulation to the TI problem in comparison with previous optimization approaches.

We also chose to define outcomes of selection criteria as discrete events. Thus, the distinction between probability of success and probability of meeting selection criteria revealed that for some selection strategies the probability of obtaining selection criteria can be very high in a few generations with small sample sizes, while their probability of success is very low. For example, from the results we see that strategy 1 with a sample size of 100 progeny per generation has a probability of meeting selection criteria of almost 1 in about four generations, but a *P*(s) of only 0.034. If the budget of an introgression program is too small to obtain a high *P*(s), such as observed in strategy 1 with *np* = 100 per generation, then it would be better to do nothing rather than to waste resources. The incredibly low *P*(s) of strategy 1 with *np* = 100 is most likely due to too few gametes being sampled each generation to observe recombination in close proximity with the *el*. Strategies 1 and 2 both reach a plateau of *P*(s) at 0.38; below that of strategy 3 which has a maximum *P*(s) of 0.52 (Fig. 3). The increase in *P*(s) of strategy 3 over the *P*(s) of strategies 1 and 2 is likely due to more selection being allowed on the *ll*; however, it is also likely to be due in part to the very strict selection of only 8 individuals for LDS irrespective of sample size inhibiting the fixation of the *ul* and thus leading to more backcross generations to find recombination on both sides of the *el*. We did not investigate differences in numbers of *ll* markers among breeding strategies, but did find that the greatest impact on *P*(s) was realized from doubling the number of markers in the *ul* (Figs. 3, 4). Reducing the size of the genetic intervals among the *ul* increases the probability of detecting double recombination events concealing NRP in the interval between adjacent *ul*. We conclude that a marker every 20 cM (Peng et al. 2014) is not sufficient to produce a high frequency of progeny with a total NRP ≤8 cM in each of the four final ILs. Herzog and Frisch (2011) found that a marker every 10 cM was optimal with respect to the distribution of recurrent parent in 10% quantiles of the final backcross generation. Our results are consistent with Herzog and Frisch (2011) because the *P*(s) with markers at 10 cM intervals are greater than 0.9 and further improvements will be incrementally small.

To define discrete events for selection criteria and success, we needed explicit mathematical definitions and notation. Probability and set theory provided the foundation for these definitions (Hoel et al. 1971). Note that if *ml* represent a random sample of *l*, then *pml/ml* = frequency of *pml* should provide an unbiased estimate of the frequency of *pl* among all loci in the genome, i.e., *E*(*pml/ml*) = *pl/l*. In practice, we know that this is not true, because marker technologies such as the Tape Array are biased toward detection of *pl*. For situations involving marker-assisted breeding for TI, where the goal is to eliminate alleles from the donor parent, such detection bias of marker technologies is desirable.

Costs associated with marker technologies were based on interviews with marker assay service providers and are likely to decline in the future. Not all marker assays provide the same number of informative markers nor the same cost per informative marker. For example, technology options might include: (A) Tape Array for about a nickel/marker/sample; (B) Genotyping by Sequencing (GBS) for about 10–15 dollars per sample to assay 1200 markers and (C) BeadChip for about 25–30 dollars per sample to assay 12,000 markers. Tape Arrays enable the breeder to select markers that will be informative, i.e., polymorphic at specific genomic sites in the two parental lines. GBS and BeadChip represent ‘batch’ processes in which some proportion of the markers will be informative. For example, in a typical cross involving elite varieties of soybean, only about 1/5 of the markers will be informative for the BeadChip technologies (Song et al. 2015).

It is important to emphasize that while the cost per marker is highly variable among technologies, the marker costs are small regardless of the technologies when compared to the costs of growing plants, sampling tissue and extracting DNA for analysis. For the Tape Array technology, it would be possible to further reduce the costs by recognizing that it is not necessary to assay any *pml* that are homozygous for the RP. Thus, the marker costs are slightly lower than we reported for all combinations of *np* and strategies.

The k strategies that incorporated dynamic decision making based on selection criteria produced greater *P*(s) than the k′ strategies that were constrained to five backcross generations. Peng et al. (2014) found strategy 2′ to be optimal in their study. Comparing strategies 5′ and 2′, we see that strategy 5′ can double the probability of success for less cost. The *P*(s) for strategy 2′ can achieve a maximum of 0.384 for a cost of $32,942, whereas strategy 5′ can achieve a *P*(s) of 0.844 for a cost of $29,245 (Supplemental Materials). Overall the greatest *P*(s) for the least cost ($10,000) was achieved with *k* = 6; however, *k* = 6 required about eight generations. Strategy 5′ achieved a *P*(s) that was almost as good in only five generations, but required a sample size of about 700 per generation and a cost of about $26,000. The results raise a question about the cost of time. Would an extra $16,000 be justified if the project were completed in three fewer generations? For organizations with continuous maize nurseries, this translates into a year.

Bringing a new product to the market before a competitor could result in a larger share of the market, while arriving to the market with a product after a competitor could incur a penalty of lost market share. Thus, the cost of the breeding strategy is not the only cost. Consider the experience with herbicide resistance: From 1996 to 2001, the proportion of soybean seed with herbicide resistance grew from 0 to 68% and slowed thereafter to about 6% for the next three years, 2% for the subsequent 3 years and has remained at about 93% since 2010 (USDA-ERS 2016). The size of the US soybean market is about $12B. If market share was 25%, the tangible costs associated with taking an extra year would be ~13% of $3B or about $400 M. Clearly, for herbicide resistance, the extra cost required to eliminate a year of development time would be justified for a project designed to introgress the event into many elite varieties.

Was the marketplace adoption of herbicide resistance a typical TI example? Probably not. The question of more appropriate forecast models that ascribe benefits (or costs) to time will be needed on a trait by trait basis and we suggest that this could be an exciting and useful topic for future research collaborations between plant breeders and agricultural economists who are already familiar with OR approaches to optimization and modeling.

In this report, we did not propose any new selection strategies, rather we used a few of the same selection strategies propose by Peng et al. (2014), but framed these in the context of a CTP model. For strategies employing LDS only 1 side of the event (i.e., 10 of the 20 *ll* markers) was evaluated per generation, the side evaluated alternated each generation. The rationale for not selecting on all *ll* markers during LDS was not explained by Peng et al. (2014), but it is reasonable to assume that they chose this strategy because the probability of observing double recombinant is extremely low in the regions immediately adjacent to the event locus. We hypothesize that other selection strategies such as those described by Herzog and Frisch (2011) will enable even better Pareto optimality in a CTP framework.

Last we would like to remind the reader of some practical considerations for planning operational TI projects. **M1** was formulated for the reproductive biology of maize, where it is possible to obtain ≈300 seeds from a cross about every 100 days. Thus, it is reasonable to obtain 1000 progeny per generation from four selected individuals per generation. In self-pollinated species such as soybean, the reproductive biology is not as facile. The number of crossed progeny attainable per soybean plant will be no more than 100 per RP plant as the number of successful pollinations per plant is usually no more than ~50. Thus, selected individual plants in any given generation will have to be used as pollen sources for crossing with multiple individuals representing the RP line. Further, selected individuals would be limited in their nicking ability with the RP, so the individual plants representing the RP would have to be planted at multiple times. Nonetheless, the parameters of the CTP model can be changed to replicate the biological reality of the crop under consideration, and uncertainty can be built into the model using distributions instead of point estimates for parameters.

### **Author contribution statement**

JC developed the analytical tools to carry out the analysis, wrote the initial manuscript drafts, and created the figures and tables. YH provided guidance for efficient software programming. LW guided development of mathematical objective functions in the framework of cost, time, and probability of success. WDB developed the mathematical model, provided interpretation of the results and was responsible for the final drafts of the manuscript.

## Electronic supplementary material

Below is the link to the electronic supplementary material.

Supplementary material 1 (DOCX 73 kb)

Supplementary material 2 (DOCX 60 kb)

Supplementary material 3 (DOCX 71 kb)
