# Supplementary material for: Systematic design for trait introgression projects
Source: Theor Appl Genet. 2017 Jun 24;130(10):1993–2004. doi: 10.1007/s00122-017-2938-9 (PMC5606951; doi:10.1007/s00122-017-2938-9)
Supplement: Supplementary file 1 — Supplementary material 1 (DOCX 73 kb) [file 122_2017_2938_MOESM1_ESM.docx]

**Systematic Design for Trait Introgression Projects**

John N Cameron

Department of Agronomy, Iowa State University, Ames, IA 50010, [jncamero@iastate.edu](mailto:jncamero@iastate.edu)

Ye Han

Lizhi Wang

William D Beavis

The following six tables provide summary data from the simulations of the strategies in **M1**. Generation is indicated by g and the number of progeny evaluated per generation is indicated by *np_g_*. P(*t’*≤g) indicates the probability that the terminal generation is reached by generation g. P(*s*) indicates cumulative probability of successfully achieving the project goals by generation g. Cost is given in US dollars, and is the expected cost of carrying out g generations for the specified strategy.

Table 1 Summary data for Strategy 1

| g | *np_g_* | P(*t’*≤g) | P(s) | Cost ($) |
| --- | --- | --- | --- | --- |
|  | 100 |  |  |  |
| 3 |  | 0.001 | 0 | 1765 |
| 4 |  | 0.52 | 0.01 | 2620 |
| 5 |  | 0.991 | 0.034 | 3475 |
| 6 |  | 0.999 | 0.034 | 4330 |
| 7 |  | 1 | 0.034 | 5185 |
|  | 200 |  |  |  |
| 3 |  | 0.005 | 0.001 | 3530 |
| 4 |  | 0.909 | 0.064 | 5240 |
| 5 |  | 1 | 0.077 | 6950 |
|  | 300 |  |  |  |
| 3 |  | 0.009 | 0 | 5295 |
| 4 |  | 0.969 | 0.125 | 7860 |
| 5 |  | 1 | 0.13 | 10425 |
|  | 400 |  |  |  |
| 3 |  | 0.012 | 0.003 | 7060 |
| 4 |  | 0.993 | 0.180 | 10480 |
| 5 |  | 1 | 0.184 | 13900 |
|  | 500 |  |  |  |
| 3 |  | 0.016 | 0.004 | 8825 |
| 4 |  | 0.997 | 0.259 | 13100 |
| 5 |  | 1 | 0.260 | 17375 |
|  | 600 |  |  |  |
| 3 |  | 0.019 | 0.004 | 10590 |
| 4 |  | 0.999 | 0.308 | 15720 |
| 5 |  | 1 | 0.309 | 20850 |
|  | 700 |  |  |  |
| 3 |  | 0.030 | 0.004 | 12355 |
| 4 |  | 1 | 0.343 | 18340 |
|  | 800 |  |  |  |
| 3 |  | 0.037 | 0.009 | 14120 |
| 4 |  | 1 | 0.346 | 20960 |
|  | 900 |  |  |  |
| 3 |  | 0.046 | 0.011 | 15885 |
| 4 |  | 1 | 0.374 | 23580 |
|  | 1000 |  |  |  |
| 3 |  | 0.044 | 0.009 | 17650 |
| 4 |  | 1 | 0.382 | 26200 |

Table 2 Summary data for Strategy 2

| g | *np_g_* | P(*t’*≤g) | P(s) | Cost ($) |
| --- | --- | --- | --- | --- |
|  | 100 |  |  |  |
| 4 |  | 0.087 | 0.006 | 2221 |
| 5 |  | 0.911 | 0.080 | 3076 |
| 6 |  | 0.998 | 0.088 | 3931 |
| 7 |  | 1 | 0.088 | 4786 |
|  | 200 |  |  |  |
| 4 |  | 0.208 | 0.027 | 4444 |
| 5 |  | 0.989 | 0.220 | 6154 |
| 6 |  | 1 | 0.223 | 7864 |
|  | 300 |  |  |  |
| 4 |  | 0.278 | 0.056 | 6685.2 |
| 5 |  | 0.995 | 0.304 | 9250.2 |
| 6 |  | 1 | 0.305 | 11815 |
|  | 400 |  |  |  |
| 4 |  | 0.330 | 0.074 | 8949.6 |
| 5 |  | 0.998 | 0.330 | 12370 |
| 6 |  | 1 | 0.331 | 15790 |
|  | 500 |  |  |  |
| 4 |  | 0.374 | 0.090 | 11276 |
| 5 |  | 1 | 0.360 | 15551 |
|  | 600 |  |  |  |
| 4 |  | 0.403 | 0.120 | 13694 |
| 5 |  | 1 | 0.361 | 18824 |
|  | 700 |  |  |  |
| 4 |  | 0.469 | 0.132 | 16201 |
| 5 |  | 1 | 0.355 | 22186 |
|  | 800 |  |  |  |
| 4 |  | 0.551 | 0.184 | 18853 |
| 5 |  | 1 | 0.364 | 25693 |
|  | 900 |  |  |  |
| 4 |  | 0.560 | 0.211 | 21595 |
| 5 |  | 1 | 0.376 | 29290 |
|  | 1000 |  |  |  |
| 4 |  | 0.684 | 0.251 | 24392 |
| 5 |  | 1 | 0.384 | 32942 |

Table 3 Summary data for Strategy 3

| g | *np_g_* | P(*t’*≤g) | P(s) | Cost ($) |
| --- | --- | --- | --- | --- |
|  | 100 |  |  |  |
| 3 |  | 0.001 | 0 | 2864.8 |
| 4 |  | 0.091 | 0.009 | 3816.2 |
| 5 |  | 0.645 | 0.074 | 4761.9 |
| 6 |  | 0.961 | 0.125 | 5701.6 |
| 7 |  | 0.998 | 0.133 | 6645.9 |
| 8 |  | 1 | 0.134 | 7595.2 |
|  | 200 |  |  |  |
| 3 |  | 0.001 | 0 | 5728.4 |
| 4 |  | 0.125 | 0.020 | 7615.1 |
| 5 |  | 0.716 | 0.155 | 9482.1 |
| 6 |  | 0.985 | 0.263 | 11336 |
| 7 |  | 1 | 0.270 | 13219 |
|  | 300 |  |  |  |
| 4 |  | 0.151 | 0.040 | 11387 |
| 5 |  | 0.738 | 0.250 | 14145 |
| 6 |  | 0.987 | 0.375 | 16920 |
| 7 |  | 1 | 0.384 | 19758 |
|  | 400 |  |  |  |
| 3 |  | 0.001 | 0 | 11447 |
| 4 |  | 0.193 | 0.063 | 15107 |
| 5 |  | 0.789 | 0.322 | 18767 |
| 6 |  | 0.993 | 0.447 | 22493 |
| 7 |  | 1 | 0.453 | 26295 |
|  | 500 |  |  |  |
| 3 |  | 0.002 | 0.001 | 14292 |
| 4 |  | 0.248 | 0.069 | 18800 |
| 5 |  | 0.807 | 0.364 | 23369 |
| 6 |  | 0.991 | 0.485 | 28060 |
| 7 |  | 1 | 0.492 | 32826 |
|  | 600 |  |  |  |
| 3 |  | 0.003 | 0.001 | 17118 |
| 4 |  | 0.320 | 0.099 | 22459 |
| 5 |  | 0.836 | 0.380 | 27966 |
| 6 |  | 0.997 | 0.506 | 33630 |
| 7 |  | 1 | 0.509 | 39355 |
|  | 700 |  |  |  |
| 3 |  | 0.003 | 0.001 | 19902 |
| 4 |  | 0.384 | 0.150 | 26049 |
| 5 |  | 0.877 | 0.438 | 32518 |
| 6 |  | 0.996 | 0.521 | 39170 |
| 7 |  | 1 | 0.524 | 45852 |
|  | 800 |  |  |  |
| 3 |  | 0.005 | 0.002 | 22652 |
| 4 |  | 0.444 | 0.164 | 29631 |
| 5 |  | 0.918 | 0.435 | 37093 |
| 6 |  | 0.998 | 0.492 | 44704 |
| 7 |  | 1 | 0.494 | 52344 |
|  | 900 |  |  |  |
| 3 |  | 0.008 | 0.001 | 25387 |
| 4 |  | 0.514 | 0.194 | 33186 |
| 5 |  | 0.934 | 0.455 | 41633 |
| 6 |  | 0.998 | 0.505 | 50213 |
| 7 |  | 1 | 0.507 | 58808 |
|  | 1000 |  |  |  |
| 3 |  | 0.013 | 0.002 | 28146 |
| 4 |  | 0.603 | 0.217 | 36794 |
| 5 |  | 0.938 | 0.424 | 46220 |
| 6 |  | 1 | 0.472 | 55763 |

Table 4 Summary data for Strategy 4

| g | *np_g_* | P(*t’*≤g) | P(s) | Cost ($) |
| --- | --- | --- | --- | --- |
|  | 100 |  |  |  |
| 4 |  | 0.317 | 0.016 | 2215 |
| 5 |  | 0.965 | 0.074 | 3520 |
| 6 |  | 1 | 0.078 | 4825 |
|  | 200 |  |  |  |
| 3 |  | 0.002 | 0.016 | 4430 |
| 4 |  | 0.753 | 0.074 | 7040 |
| 5 |  | 0.999 | 0.078 | 9650 |
| 6 |  | 1 | 0.078 | 12260 |
|  | 300 |  |  |  |
| 3 |  | 0.005 | 0.002 | 6645 |
| 4 |  | 0.876 | 0.257 | 10560 |
| 5 |  | 1 | 0.294 | 14475 |
|  | 400 |  |  |  |
| 3 |  | 0.007 | 0.002 | 8860 |
| 4 |  | 0.946 | 0.442 | 14080 |
| 5 |  | 1 | 0.471 | 19300 |
|  | 500 |  |  |  |
| 3 |  | 0.009 | 0.005 | 11075 |
| 4 |  | 0.970 | 0.586 | 17600 |
| 5 |  | 1 | 0.605 | 24125 |
|  | 600 |  |  |  |
| 3 |  | 0.009 | 0.006 | 13290 |
| 4 |  | 0.984 | 0.676 | 21120 |
| 5 |  | 1 | 0.688 | 28950 |
|  | 700 |  |  |  |
| 3 |  | 0.013 | 0.008 | 15505 |
| 4 |  | 0.993 | 0.752 | 24640 |
| 5 |  | 1 | 0.759 | 33775 |
|  | 800 |  |  |  |
| 3 |  | 0.015 | 0.012 | 17720 |
| 4 |  | 0.996 | 0.803 | 28160 |
| 5 |  | 1 | 0.807 | 38600 |
|  | 900 |  |  |  |
| 3 |  | 0.015 | 0.012 | 19935 |
| 4 |  | 0.996 | 0.821 | 31680 |
| 5 |  | 1 | 0.825 | 43425 |
|  | 1000 |  |  |  |
| 3 |  | 0.023 | 0.017 | 22150 |
| 4 |  | 1 | 0.846 | 35200 |

Table 5 Summary data for Strategy 5

| g | *np_g_* | P(*t’*≤g) | P(s) | Cost ($) |
| --- | --- | --- | --- | --- |
|  | 100 |  |  |  |
| 4 |  | 0.043 | 0.006 | 2670.1 |
| 5 |  | 0.8195 | 0.145 | 3975.1 |
| 6 |  | 0.994 | 0.172 | 5280.1 |
| 7 |  | 1 | 0.173 | 6585.1 |
|  | 200 |  |  |  |
| 4 |  | 0.1205 | 0.049 | 5344.4 |
| 5 |  | 0.9595 | 0.427 | 7954.4 |
| 6 |  | 1 | 0.445 | 10564 |
|  | 300 |  |  |  |
| 4 |  | 0.178 | 0.107 | 8026.9 |
| 5 |  | 0.9795 | 0.617 | 11942 |
| 6 |  | 1 | 0.634 | 15857 |
|  | 400 |  |  |  |
| 4 |  | 0.21 | 0.143 | 10791 |
| 5 |  | 0.989 | 0.729 | 16011 |
| 6 |  | 1 | 0.738 | 21231 |
|  | 500 |  |  |  |
| 4 |  | 0.275 | 0.213 | 13669 |
| 5 |  | 0.994 | 0.782 | 20194 |
| 6 |  | 1 | 0.788 | 26719 |
|  | 600 |  |  |  |
| 4 |  | 0.322 | 0.258 | 16752 |
| 5 |  | 0.995 | 0.820 | 24582 |
| 6 |  | 1 | 0.825 | 32412 |
|  | 700 |  |  |  |
| 4 |  | 0.376 | 0.308 | 20110 |
| 5 |  | 0.996 | 0.844 | 29245 |
| 6 |  | 1 | 0.848 | 38380 |
|  | 800 |  |  |  |
| 4 |  | 0.473 | 0.399 | 23523 |
| 5 |  | 0.998 | 0.847 | 33963 |
| 6 |  | 1 | 0.849 | 44403 |
|  | 900 |  |  |  |
| 4 |  | 0.551 | 0.465 | 27439 |
| 5 |  | 1 | 0.851 | 39184 |
|  | 1000 |  |  |  |
| 4 |  | 0.628 | 0.531 | 31159 |
| 5 |  | 1 | 0.855 | 44209 |

Table 6 Summary data for Strategy 6

| g | *np_g_* | P(*t’*≤g) | P(s) | Cost ($) |
| --- | --- | --- | --- | --- |
|  | 100 |  |  |  |
| 4 |  | 0.006 | 0.004 | 5615.4 |
| 5 |  | 0.118 | 0.080 | 7010.3 |
| 6 |  | 0.434 | 0.290 | 8398.1 |
| 7 |  | 0.761 | 0.500 | 9790.1 |
| 8 |  | 0.924 | 0.618 | 11188 |
| 9 |  | 0.983 | 0.666 | 12591 |
| 10 |  | 0.998 | 0.679 | 13996 |
| 11 |  | 1 | 0.680 | 15400 |
|  | 200 |  |  |  |
| 4 |  | 0.033 | 0.030 | 11213 |
| 5 |  | 0.254 | 0.222 | 13973 |
| 6 |  | 0.642 | 0.564 | 16725 |
| 7 |  | 0.898 | 0.781 | 19504 |
| 8 |  | 0.981 | 0.860 | 22304 |
| 9 |  | 0.996 | 0.874 | 25112 |
| 10 |  | 0.998 | 0.876 | 27921 |
| 11 |  | 1 | 0.878 | 30731 |
|  | 300 |  |  |  |
| 4 |  | 0.093 | 0.084 | 16784 |
| 5 |  | 0.469 | 0.412 | 20883 |
| 6 |  | 0.833 | 0.753 | 24995 |
| 7 |  | 0.964 | 0.874 | 29178 |
| 8 |  | 0.993 | 0.902 | 33386 |
| 9 |  | 1 | 0.907 | 37600 |
|  | 400 |  |  |  |
| 4 |  | 0.162 | 0.139 | 22309 |
| 5 |  | 0.641 | 0.576 | 27733 |
| 6 |  | 0.934 | 0.853 | 33249 |
| 7 |  | 0.995 | 0.912 | 38849 |
| 8 |  | 1 | 0.916 | 44465 |
|  | 500 |  |  |  |
| 3 |  | 0.002 | 0.002 | 21036 |
| 4 |  | 0.275 | 0.224 | 27793 |
| 5 |  | 0.784 | 0.681 | 34565 |
| 6 |  | 0.973 | 0.862 | 41499 |
| 7 |  | 0.997 | 0.885 | 48510 |
| 8 |  | 1 | 0.887 | 55534 |
|  | 600 |  |  |  |
| 3 |  | 0.001 | 0.001 | 25207 |
| 4 |  | 0.394 | 0.322 | 33229 |
| 5 |  | 0.863 | 0.757 | 41379 |
| 6 |  | 0.988 | 0.876 | 49746 |
| 7 |  | 0.998 | 0.886 | 58170 |
| 8 |  | 1 | 0.888 | 66599 |
|  | 700 |  |  |  |
| 3 |  | 0.004 | 0.003 | 29359 |
| 4 |  | 0.499 | 0.418 | 38658 |
| 5 |  | 0.928 | 0.813 | 48208 |
| 6 |  | 0.997 | 0.879 | 57997 |
| 7 |  | 1 | 0.882 | 67831 |
|  | 800 |  |  |  |
| 3 |  | 0.008 | 0.006 | 33473 |
| 4 |  | 0.629 | 0.536 | 44039 |
| 5 |  | 0.954 | 0.826 | 55010 |
| 6 |  | 0.998 | 0.869 | 66222 |
| 7 |  | 1 | 0.871 | 77460 |
|  | 900 |  |  |  |
| 3 |  | 0.013 | 0.012 | 37572 |
| 4 |  | 0.724 | 0.616 | 49425 |
| 5 |  | 0.979 | 0.852 | 61832 |
| 6 |  | 1 | 0.872 | 74459 |
|  | 1000 |  |  |  |
| 3 |  | 0.015 | 0.012 | 41648 |
| 4 |  | 0.806 | 0.692 | 54776 |
| 5 |  | 0.992 | 0.86 | 68622 |
| 6 |  | 1 | 0.868 | 82662 |
