# Supplementary material for: Systematic design for trait introgression projects
Source: Theor Appl Genet. 2017 Jun 24;130(10):1993–2004. doi: 10.1007/s00122-017-2938-9 (PMC5606951; doi:10.1007/s00122-017-2938-9)
Supplement: Supplementary file 2 — Supplementary material 2 (DOCX 60 kb) [file 122_2017_2938_MOESM2_ESM.docx]

**Systematic Design for Trait Introgression Projects**

John N Cameron

Department of Agronomy, Iowa State University, Ames, IA 50010, [jncamero@iastate.edu](mailto:jncamero@iastate.edu)

Ye Han

Lizhi Wang

William D Beavis

**Appendix 1. Simulation**

*Transmission Genetics.* Maize is a diploid species with a genome size of around 1,788 cM (Fu, Wen et al. 2006) distributed among ten independently segregating chromosomes. Thus, each individual’s genome was modeled as matrix consisting of 1800 rows and two columns. Each row represents a genetic locus and each column represents one of the two alleles at each locus. The 1800 loci were divided into ten groups of 180 to represent ten linkage groups.

The event locus (*el*) was arbitrarily designated as locus 90 on linkage group 1. The Donor genome was modeled as an 1800x2 matrix composed almost entirely of 0s with only the two entries in row 90 (*el*) having a value of 1. For notational purposes, we use $\boldsymbol{\vdots}$ to indicate that we are not at the end of a linkage group. To illustrate, the carrier chromosome of the Donor parent from locus 88 to 92 is represented as:

$$\left[ \begin{aligned} \begin{matrix} \vdots& \vdots\end{matrix} \\ \begin{matrix} 0 & 0 \end{matrix} \\ \begin{matrix} 0 & 0 \end{matrix} \\ \begin{matrix} 1 & 1 \end{matrix} \\ \begin{matrix} 0 & 0 \end{matrix} \\ \begin{matrix} 0 & 0 \end{matrix} \\ \begin{matrix} \vdots& \vdots\end{matrix} \end{aligned} \right]$$

The Recurrent parent (RP) genome was modeled as an 1800x2 matrix composed almost entirely of 1s with only the entries in row 90 both set to 0, indicating the absence of the desirable allele at the *el*. The carrier chromosome of the RP from locus 88 to 92 is represented as:

$$\left[ \begin{aligned} \begin{matrix} \vdots& \vdots\end{matrix} \\ \begin{matrix} 1 & 1 \end{matrix} \\ \begin{matrix} 1 & 1 \end{matrix} \\ \begin{matrix} 0 & 0 \end{matrix} \\ \begin{matrix} 1 & 1 \end{matrix} \\ \begin{matrix} 1 & 1 \end{matrix} \\ \begin{matrix} \vdots& \vdots\end{matrix} \end{aligned} \right]$$

To simulate meiosis for each generation of backcrossing, recombinant gametes from each individual were created using a recombination frequency of 0.01 for adjacent marker loci within each chromosome; $1-e^{\left( -2*0.01 \right)}=0.009900663 \approx0.01$ (Haldane, 1919). Between linkage groups a value of 0.5 was specified as the frequency with which a chromosome would be present in the gamete; this is in accordance with the Mendelian law of independent assortment. The values corresponding to the recombination rates between each pair of adjacent loci within chromosomes and the probability of chromosome assignments to gametes were assembled in a vector $\boldsymbol{c}$. A vector $\boldsymbol{r}$ of identical length to $\boldsymbol{c}$ containing uniformly distributed random numbers between 0 and 1 was created, and each entry was compared to its corresponding entry in $\boldsymbol{c}$. During the meiosis stage of the simulation, recombination between homologous chromosomes occurred when an entry from the random number generator was less than it’s corresponding entry in the recombination rate vector; i.e. if $r_{i} \leq c_{i}$. The gamete composition with respect to which of the recombinant chromosomes was assigned to a gamete was determined in a similar way; if the entry in $\boldsymbol{r}$ corresponding to the position between linkage groups in the matrix notation was less than 0.5, the homologous chromosomes switched column positions in the genome matrix. To illustrate, consider the following example where $\ldots$ is used to indicate the end of a linkage group.

Example

$\boldsymbol{r}=\left[ \begin{matrix} \begin{matrix} \vdots\\ 0.0855 \end{matrix} \\ 0.2625 \\ \begin{matrix} 0.4512 \\ 0.0043 \\ \begin{matrix} 0.9298 \\ \vdots\end{matrix} \end{matrix} \end{matrix} \right]$, $\boldsymbol{c}=\left[ \begin{matrix} \begin{matrix} \begin{matrix} \vdots\\ 0.01 \end{matrix} \\ 0.01 \\ 0.50 \end{matrix} \\ 0.01 \\ \begin{matrix} 0.01 \\ \vdots\end{matrix} \end{matrix} \right] , \boldsymbol{n}=\left[ \begin{matrix} \begin{matrix} \begin{matrix} \vdots& \vdots\end{matrix} \\ \begin{matrix} 1 & 0 \end{matrix} \end{matrix} \\ \begin{matrix} \begin{matrix} 1 & 0 \end{matrix} \\ \begin{matrix} 1 & 0 \end{matrix} \\ \begin{matrix} \ldots\\ \begin{matrix} \begin{matrix} 1 & 0 \end{matrix} \\ \begin{matrix} \begin{matrix} 1 & 0 \end{matrix} \\ \begin{matrix} \begin{matrix} 1 & 0 \end{matrix} \\ \begin{matrix} \vdots& \vdots\end{matrix} \end{matrix} \end{matrix} \end{matrix} \end{matrix} \end{matrix} \end{matrix} \right]$ $\overset{\Rightarrow}{\boldsymbol{meiosis}}$ $\boldsymbol{g}=\left[ \begin{matrix} \begin{matrix} \begin{matrix} \vdots& \vdots\end{matrix} \\ \begin{matrix} 1 & 0 \end{matrix} \end{matrix} \\ \begin{matrix} \begin{matrix} 1 & 0 \end{matrix} \\ \begin{matrix} 1 & 0 \end{matrix} \\ \begin{matrix} \ldots\\ \begin{matrix} \begin{matrix} 1 & 0 \end{matrix} \\ \begin{matrix} \begin{matrix} 0 & 1 \end{matrix} \\ \begin{matrix} \begin{matrix} 0 & 1 \end{matrix} \\ \begin{matrix} \vdots& \vdots\end{matrix} \end{matrix} \end{matrix} \end{matrix} \end{matrix} \end{matrix} \end{matrix} \right]$

Details for accomplishing the example outcome consist of two steps that are not consistent with meiosis, but generate the same result with less computational effort than steps that are consistent with meiosis:

1. Step 1

$\left[ \begin{matrix} \begin{matrix} \vdots\\ 0.0855 \end{matrix} \\ 0.2625 \\ \begin{matrix} 0.4512 \\ 0.0043 \\ \begin{matrix} 0.9298 \\ \vdots\end{matrix} \end{matrix} \end{matrix} \right] \begin{matrix} \begin{matrix} \vdots\\ > \\ > \end{matrix} \\ \boldsymbol{\leq} \\ \begin{matrix} \leq\\ \begin{matrix} > \\ \vdots\end{matrix} \end{matrix} \end{matrix} \left[ \begin{matrix} \begin{matrix} \begin{matrix} \vdots\\ 0.01 \end{matrix} \\ 0.01 \\ 0.50 \end{matrix} \\ 0.01 \\ \begin{matrix} 0.01 \\ \vdots\end{matrix} \end{matrix} \right] \left[ \begin{matrix} \begin{matrix} \begin{matrix} \vdots& \vdots\end{matrix} \\ \begin{matrix} 1 & 0 \end{matrix} \end{matrix} \\ \begin{matrix} \begin{matrix} 1 & 0 \end{matrix} \\ \begin{matrix} 1 & 0 \end{matrix} \\ \begin{matrix} \boldsymbol{\ldots} \\ \begin{matrix} \begin{matrix} \boldsymbol{1} & \boldsymbol{0} \end{matrix} \\ \begin{matrix} \begin{matrix} 1 & 0 \end{matrix} \\ \begin{matrix} \begin{matrix} 1 & 0 \end{matrix} \\ \begin{matrix} \vdots& \vdots\end{matrix} \end{matrix} \end{matrix} \end{matrix} \end{matrix} \end{matrix} \end{matrix} \right]$ $\overset{\Rightarrow}{\begin{aligned} \boldsymbol{Chromosome} \\ \boldsymbol{segregation} \end{aligned}}$ $\boldsymbol{g}= \left[ \begin{matrix} \begin{matrix} \begin{matrix} \begin{matrix} \begin{matrix} \vdots\\ \begin{matrix} 1 & 0 \end{matrix} \end{matrix} \\ \begin{matrix} 1 & 0 \end{matrix} \end{matrix} \\ \begin{matrix} 1 & 0 \end{matrix} \end{matrix} \\ \boldsymbol{\ldots} \\ \begin{matrix} 0 & 1 \end{matrix} \end{matrix} \\ \begin{matrix} \begin{matrix} 0 & 1 \end{matrix} \\ \begin{matrix} 0 & 1 \end{matrix} \\ \vdots\end{matrix} \end{matrix} \right]$

2. Step 2

$\left[ \begin{matrix} \begin{matrix} \vdots\\ 0.0855 \end{matrix} \\ 0.2625 \\ \begin{matrix} 0.4512 \\ 0.0043 \\ \begin{matrix} 0.9298 \\ \vdots\end{matrix} \end{matrix} \end{matrix} \right] \begin{matrix} \begin{matrix} \vdots\\ > \\ > \end{matrix} \\ \boldsymbol{\leq} \\ \begin{matrix} \boldsymbol{\leq} \\ \begin{matrix} > \\ \vdots\end{matrix} \end{matrix} \end{matrix} \left[ \begin{matrix} \begin{matrix} \begin{matrix} \vdots\\ 0.01 \end{matrix} \\ 0.01 \\ 0.50 \end{matrix} \\ 0.01 \\ \begin{matrix} 0.01 \\ \vdots\end{matrix} \end{matrix} \right] \left[ \begin{matrix} \begin{matrix} \begin{matrix} \vdots& \vdots\end{matrix} \\ \begin{matrix} 1 & 0 \end{matrix} \end{matrix} \\ \begin{matrix} \begin{matrix} 1 & 0 \end{matrix} \\ \begin{matrix} 1 & 0 \end{matrix} \\ \begin{matrix} \ldots\\ \begin{matrix} \begin{matrix} \boldsymbol{0} & \boldsymbol{1} \end{matrix} \\ \begin{matrix} \begin{matrix} \boldsymbol{0} & \boldsymbol{1} \end{matrix} \\ \begin{matrix} \begin{matrix} 0 & 1 \end{matrix} \\ \begin{matrix} \vdots& \vdots\end{matrix} \end{matrix} \end{matrix} \end{matrix} \end{matrix} \end{matrix} \end{matrix} \right]$ $\overset{\Rightarrow}{\boldsymbol{Recombination}}$ $\boldsymbol{g}= \left[ \begin{matrix} \begin{matrix} \begin{matrix} \begin{matrix} \begin{matrix} \vdots\\ \begin{matrix} 1 & 0 \end{matrix} \end{matrix} \\ \begin{matrix} 1 & 0 \end{matrix} \end{matrix} \\ \begin{matrix} 1 & 0 \end{matrix} \end{matrix} \\ \ldots\\ \begin{matrix} 0 & 1 \end{matrix} \end{matrix} \\ \begin{matrix} \begin{matrix} 1 & 0 \end{matrix} \\ \begin{matrix} 1 & 0 \end{matrix} \\ \vdots\end{matrix} \end{matrix} \right]$

Thus, each of the two vectors in the output matrix ($\boldsymbol{g}$) represents a gamete from an individual. A random number generator is used once again to determine which of the gametes the backcross progeny will receive. If the value of the random number (*u*) is less than 0.5, the left vector is selected, while if it is greater than 0.5 the right vector is selected.

i.e. $u=0.2679\leq0.5$0

$$BC gametes reccurent parent gamete$$

$$\begin{matrix} \begin{matrix} \begin{matrix} \vdots& \vdots\end{matrix} \\ \begin{matrix} 1 & 0 \end{matrix} \end{matrix} \\ \begin{matrix} 1 & 0 \end{matrix} \\ \begin{matrix} \begin{matrix} 1 & 0 \end{matrix} \\ \begin{matrix} \vdots& \vdots\end{matrix} \\ \begin{matrix} \begin{matrix} 0 & 1 \end{matrix} \\ \begin{matrix} 1 & 0 \end{matrix} \\ \begin{matrix} \begin{matrix} 1 & 0 \end{matrix} \\ \begin{matrix} \vdots& \vdots\end{matrix} \end{matrix} \end{matrix} \end{matrix} \end{matrix} \begin{matrix} \vdots\\ 1 \\ \begin{matrix} 1 \\ 1 \\ \begin{matrix} \ldots\\ 1 \\ \begin{matrix} 1 \\ 1 \\ \vdots\end{matrix} \end{matrix} \end{matrix} \end{matrix}$$

$$\boldsymbol{new BC progeny}$$

$$\left[ \begin{matrix} \begin{matrix} \vdots& \vdots\\ 1 & 1 \\ 1 & 1 \end{matrix} \\ \begin{matrix} 1 & 1 \end{matrix} \\ \begin{matrix} \begin{matrix} \cdots& \cdots\end{matrix} \\ \begin{matrix} 0 & 1 \end{matrix} \\ \begin{matrix} \begin{matrix} 1 & 1 \end{matrix} \\ \begin{matrix} 1 & 1 \end{matrix} \\ \begin{matrix} \vdots& \vdots\end{matrix} \end{matrix} \end{matrix} \end{matrix} \right]$$

The 1800x2 matrix for each individual in a generation indicates the loci with polymorphic alleles (*pl*), as described in the methods section. To reiterate, selection was not carried out on *pl*, but rather on a set of marker loci with polymorphic alleles (*pml*) making up a subset of *pl*, for each individual. The *pml* are further broken down into 3 groups: 1 marker locus designates the *el*, 20 loci (*ll*) flanking the *el* (10 on each side) are used for LDS, and 98 or 187 loci (*ul*) spread across the genome are used for RPS. The sets of *pl* for selected individuals in terminal generations were retained to calculate the efficacy of each strategy.

*Selection Criteria.* ES was implemented by evaluating the *el* (i.e. the 90^th^ entry) of a simulated “gamete” vector inherited by each progeny in each BC generation. To illustrate, consider loci 88 – 92 of the carrier chromosome for 2 BC_1_F_1_ individuals:

$individual 1 individual 2$

$$\left[ \begin{aligned} \begin{matrix} \vdots& \vdots\end{matrix} \\ \begin{matrix} 0 & 1 \end{matrix} \\ \begin{matrix} 0 & 1 \end{matrix} \\ \begin{matrix} \boldsymbol{1} & 0 \end{matrix} \\ \begin{matrix} 0 & 1 \end{matrix} \\ \begin{matrix} 1 & 1 \end{matrix} \\ \begin{matrix} \vdots& \vdots\end{matrix} \end{aligned} \right] \left[ \begin{aligned} \begin{matrix} \vdots& \vdots\end{matrix} \\ \begin{matrix} 0 & 1 \end{matrix} \\ \begin{matrix} 1 & 1 \end{matrix} \\ \begin{matrix} \boldsymbol{0} & 0 \end{matrix} \\ \begin{matrix} 1 & 1 \end{matrix} \\ \begin{matrix} 1 & 1 \end{matrix} \\ \begin{matrix} \vdots& \vdots\end{matrix} \end{aligned} \right]$$

Individual 1 carries the desirable allele at the *el* and is selected, whereas individual 2 does not carry the desirable allele and is culled.

Because double recombination events are very rare it is often necessary to alternate LDS every backcross generation. This is accomplished by developing a Foreground Region (FR) score for each individual, calculated using a weighting vector (***f***) with a length of 10 entries. The *ll* closest to the *el* received the largest weight and marker weights decrease with distance from the *el*. Let **x** be a vector containing the alleles at *ll* on one side of the *el* for an individual. The FR score for that individual in the backcross population is thus calculated as:

$$\sum_{i}^{10} f_{i}*x_{i}$$

For example, consider four progeny in a backcross generation with the desirable allele at the *el*. Also, consider the vector ***f*** with relative weights, i.e, scores assigned to each *x* on each side of the *el*. Based on each individual’s FR score, individuals 1 and 3 are selected to advance to the next generation of backcrossing. The side being evaluated in LDS alternates every generation unless all *ll* on one side of the *el* become fixed for the RP. If all of the selected individuals in a generation are fixed for the desirable alleles in the FR of one side of the *el*, the simulation will not carry out LDS on that side of the *el* again, and will instead return to evaluating the *ll* of the side that is not completely fixed for the RP.

$FR score vector \left( f \right) individual 1 individual 2 individual 3 individual 4$ $\left[ \begin{matrix} \begin{matrix} \begin{matrix} \begin{matrix} \begin{matrix} 1/55 \\ 2/55 \\ 3/55 \end{matrix} \\ 4/55 \\ 5/55 \end{matrix} \\ 6/55 \\ 7/55 \end{matrix} \\ 8/55 \\ 9/55 \end{matrix} \\ 10/55 \\ 0 \end{matrix} \right]$ $\begin{matrix} \begin{matrix} \begin{matrix} \vdots& \vdots\end{matrix} \\ \begin{matrix} \begin{matrix} 1 & 1 \end{matrix} \\ \begin{matrix} 1 & 1 \end{matrix} \end{matrix} \end{matrix} \\ \begin{matrix} 1 & 1 \end{matrix} \\ \begin{matrix} \begin{matrix} 1 & 1 \end{matrix} \\ \begin{matrix} 1 & 1 \end{matrix} \\ \begin{matrix} \begin{matrix} 1 & 1 \end{matrix} \\ \begin{matrix} 1 & 1 \end{matrix} \\ \begin{matrix} \begin{matrix} 0 & 1 \end{matrix} \\ \begin{matrix} 0 & 1 \end{matrix} \\ \begin{matrix} \begin{matrix} 0 & 1 \end{matrix} \\ \begin{matrix} 1 & 0 \end{matrix} \\ \begin{matrix} \vdots& \vdots\end{matrix} \end{matrix} \end{matrix} \end{matrix} \end{matrix} \end{matrix}$ $\begin{matrix} \begin{matrix} \begin{matrix} \vdots& \vdots\end{matrix} \\ \begin{matrix} \begin{matrix} 1 & 1 \end{matrix} \\ \begin{matrix} 1 & 1 \end{matrix} \end{matrix} \end{matrix} \\ \begin{matrix} 1 & 1 \end{matrix} \\ \begin{matrix} \begin{matrix} 0 & 1 \end{matrix} \\ \begin{matrix} 0 & 1 \end{matrix} \\ \begin{matrix} \begin{matrix} 0 & 1 \end{matrix} \\ \begin{matrix} 0 & 1 \end{matrix} \\ \begin{matrix} \begin{matrix} 0 & 1 \end{matrix} \\ \begin{matrix} 0 & 1 \end{matrix} \\ \begin{matrix} \begin{matrix} 0 & 1 \end{matrix} \\ \begin{matrix} 1 & 0 \end{matrix} \\ \begin{matrix} \vdots& \vdots\end{matrix} \end{matrix} \end{matrix} \end{matrix} \end{matrix} \end{matrix}$ $\begin{matrix} \begin{matrix} \begin{matrix} \vdots& \vdots\end{matrix} \\ \begin{matrix} \begin{matrix} 1 & 1 \end{matrix} \\ \begin{matrix} 1 & 1 \end{matrix} \end{matrix} \end{matrix} \\ \begin{matrix} 1 & 1 \end{matrix} \\ \begin{matrix} \begin{matrix} 1 & 1 \end{matrix} \\ \begin{matrix} 1 & 1 \end{matrix} \\ \begin{matrix} \begin{matrix} 0 & 1 \end{matrix} \\ \begin{matrix} 0 & 1 \end{matrix} \\ \begin{matrix} \begin{matrix} 0 & 1 \end{matrix} \\ \begin{matrix} 0 & 1 \end{matrix} \\ \begin{matrix} \begin{matrix} 0 & 1 \end{matrix} \\ \begin{matrix} 1 & 0 \end{matrix} \\ \begin{matrix} \vdots& \vdots\end{matrix} \end{matrix} \end{matrix} \end{matrix} \end{matrix} \end{matrix}$ $\begin{matrix} \begin{matrix} \begin{matrix} \vdots& \vdots\end{matrix} \\ \begin{matrix} \begin{matrix} 1 & 1 \end{matrix} \\ \begin{matrix} 1 & 1 \end{matrix} \end{matrix} \end{matrix} \\ \begin{matrix} 0 & 1 \end{matrix} \\ \begin{matrix} \begin{matrix} 0 & 1 \end{matrix} \\ \begin{matrix} 0 & 1 \end{matrix} \\ \begin{matrix} \begin{matrix} 0 & 1 \end{matrix} \\ \begin{matrix} 0 & 1 \end{matrix} \\ \begin{matrix} \begin{matrix} 0 & 1 \end{matrix} \\ \begin{matrix} 0 & 1 \end{matrix} \\ \begin{matrix} \begin{matrix} 0 & 1 \end{matrix} \\ \begin{matrix} 1 & 0 \end{matrix} \\ \begin{matrix} \vdots& \vdots\end{matrix} \end{matrix} \end{matrix} \end{matrix} \end{matrix} \end{matrix}$

Event locus

individual 1 score :$0*\frac{10}{55}+0*\frac{9}{55}+0*\frac{8}{55}+0*\frac{7}{55}+\boldsymbol{1}*\frac{6}{55}+\boldsymbol{1}*\frac{5}{55}+\boldsymbol{1}*\frac{4}{55}+\boldsymbol{1}*\frac{3}{55}+\boldsymbol{1}*\frac{2}{55}+\boldsymbol{1}*\frac{1}{55}=$ $\frac{\boldsymbol{28}}{\boldsymbol{55}}$

individual 2 score : $0*\frac{10}{55}+0*\frac{9}{55}+0*\frac{8}{55}+0*\frac{7}{55}+0*\frac{6}{55}+0*\frac{5}{55}+0*\frac{4}{55}+\boldsymbol{1}*\frac{3}{55}+\boldsymbol{1}*\frac{2}{55}+\boldsymbol{1}*\frac{1}{55}= \frac{\boldsymbol{6}}{\boldsymbol{55}}$

individual 3 score : $0*\frac{10}{55}+0*\frac{9}{55}+0*\frac{8}{55}+0*\frac{7}{55}+0*\frac{6}{55}+\boldsymbol{1}*\frac{5}{55}+\boldsymbol{1}*\frac{4}{55}+\boldsymbol{1}*\frac{3}{55}+\boldsymbol{1}*\frac{2}{55}+\boldsymbol{1}*\frac{1}{55}=\frac{\boldsymbol{15}}{\boldsymbol{55}}$

individual 4 score : $0*\frac{10}{55}+0*\frac{9}{55}+0*\frac{8}{55}+0*\frac{7}{55}+0*\frac{6}{55}+0*\frac{5}{55}+0*\frac{4}{55}+0*\frac{3}{55}+\boldsymbol{1}*\frac{2}{55}+\boldsymbol{1}*\frac{1}{55}=$ $\frac{\boldsymbol{3}}{\boldsymbol{55}}$

RPS was implemented by evaluating a subset of *pml* consisting either 98 or 187 loci spread across the genome (as described in the methods section), referred to as *ul*. Let **ul** indicate a matrix of genotypes at *ul* for an individual with 98 *ul*. The score for each individual is thus computed as the sum of *ul* with RP alleles:

$$\sum_{i=i}^{2} \sum_{j=1}^{98} {ul}_{ij}$$

**References**

Haldane J (1919) The combination of linkage values and the calculation of distances between the loci of linked factors. J Genet 8:299-309

Fu Y, Wen T-J, Ronin YI, Chen HD, Guo L, Mester DI, Yang Y, Lee M, Korol AB, Ashlock DA (2006) Genetic

dissection of intermated recombinant inbred lines using a new genetic map of maize. Genetics 174:1671-1683
