# Supplementary material for: Systematic design for trait introgression projects
Source: Theor Appl Genet. 2017 Jun 24;130(10):1993–2004. doi: 10.1007/s00122-017-2938-9 (PMC5606951; doi:10.1007/s00122-017-2938-9)
Supplement: Supplementary file 3 — Supplementary material 3 (DOCX 71 kb) [file 122_2017_2938_MOESM3_ESM.docx]

**Systematic Design for Trait Introgression Projects**

John N Cameron

Department of Agronomy, Iowa State University, Ames, IA 50010, [jncamero@iastate.edu](mailto:jncamero@iastate.edu)

Ye Han

Lizhi Wang

William D Beavis

**Appendix 2: Recombination Model**

We investigated the average number of recombinations occurring per linkage group during the formation of the non-recurrent parent (NRP) gametes during each backcross to ensure our simulation model was working properly. We found on average that ~1.8 recombinations were occuring per linkage group, per round of meiosis. This confirmed that the recombination structure of our model was working as intended.

We then decided to look at the frequency distribution of: (a) the numbers of recombination events that occured in all NRP gametes, and (b) the number of recombination events that occurred in formation of the gametes inherited by selected individuals. To accomplish (a), we recorded the number of recombinations that occurred on a carrier and a non-carrier linkage group during the formation of each NRP gamete inherited by each individual, in each generation. For (b), we recorded the number of recombinations that had occurred on each (carrier and non-carrier) of two NRP derived linkage groups in each selected individual.

We created 4 histograms showing the frequencies of the different numbers of recombination observed on the 2 linkage groups for (a) all individuals across the entire simulation, and (b) selected individuals only. We expected the histograms showing the frequencies of numbers of recombination across the entire simulation progeny size for the 2 linkage groups to be identical, and they were. For selected individuals, the histogram showing the frequency of number of recombinations on the non-carrier linkage group was nearly identical to the histogram showing the number of recombinations for all individuals on the same linkage group. For the carrier chromosome, the histogram showing recombination frequency for all individuals was very different than the histogram for selected individuals only. Individuals with 0 or 1 recombination event were less likely to be found in the selected individuals than in the overall progeny. Individuals with 2 or more recombination events were much more likely to be found in the selected progeny than in the progeny overall. For example, there was a 34.58% increase in the frequency of individuals that had 3 recombination events on chromosome 1 in the selected progeny compared with the overall progeny. There was a 57.33% increase in the frequency of individuals with 4 recombinations in the selected progeny compared with the overall progeny.

| **Selected progeny only** |  |  |  |  |  |  |  |  |  |  |
| --- | --- | --- | --- | --- | --- | --- | --- | --- | --- | --- |
| No. of recombinations | 0 | 1 | 2 | 3 | 4 | 5 | 6 | 7 | 8 | 9 |
| Frequency | 0.07085 | 0.24645 | 0.29265 | 0.2152 | 0.11185 | 0.04355 | 0.0142 | 0.0041 | 0.0009 | 0.00025 |
| **All progeny** |  |  |  |  |  |  |  |  |  |  |
| No. of recombinations | 0 | 1 | 2 | 3 | 4 | 5 | 6 | 7 | 8 | 9 |
| Frequency | 0.16547 | 0.29936 | 0.26919 | 0.15991 | 0.071093 | 0.025352 | 0.0073369 | 0.0018104 | 0.00040801 | 7.66E-05 |

Table1 Frequency of number of recombination events occurring on the carrier linkage group, per round of meiosis, for (a) all individuals, and (b) selected individuals only

Figure 1 Frequency of number of recombination events occurring on the carrier linkage group, per round of meiosis, for all individuals

Figure 2 Frequency of number of recombination events occurring on the carrier linkage group, per round of meiosis, for selected individuals only

Figure 3 Frequency of number of recombination events occurring on a non-carrier linkage group, per round of meiosis, for all individuals

Figure 4 Frequency of number of recombination events occurring on a non-carrier linkage group, per round of meiosis, for selected individuals only
